# Biota from the coastal wetlands of Praia da Vitória (Terceira, Azores, Portugal): Part 1 - Arthropods

**DOI:** 10.3897/BDJ.6.e27194

**Published:** 2018-07-25

**Authors:** Paulo Alexandre Vieira Borges, Rosalina Gabriel, César M.M. Pimentel, Mariana R. Brito, Artur Raposo Moniz Serrano, Luís Carlos Fonseca Crespo, Volker Assing, Peter Stüben, Simone Fattorini, António Onofre Soares, Enésima P. Mendonça, Elisabete Nogueira

**Affiliations:** 1 CE3C – Centre for Ecology, Evolution and Environmental Changes / Azorean Biodiversity Group and Universidade dos Açores, Angra do Heroísmo, Azores, Portugal CE3C – Centre for Ecology, Evolution and Environmental Changes / Azorean Biodiversity Group and Universidade dos Açores Angra do Heroísmo, Azores Portugal; 2 LIFE CWR – LIFE project “Ecological Restoration and Conservation of Praia da Vitória Coastal Wet Green Infrastructures”, Praia da Vitória, Azores, Portugal LIFE CWR – LIFE project “Ecological Restoration and Conservation of Praia da Vitória Coastal Wet Green Infrastructures” Praia da Vitória, Azores Portugal; 3 Departamento de Biologia Animal/, Faculdade de Ciências, Universidade de Lisboa, Lisboa, Portugal Departamento de Biologia Animal/, Faculdade de Ciências, Universidade de Lisboa Lisboa Portugal; 4 Biodiversity Research Institute UB, Departament Biologia Animal, Universitat de Barcelona, Av. Diagonal 645, E-08028, Barcelona, Spain Biodiversity Research Institute UB, Departament Biologia Animal, Universitat de Barcelona, Av. Diagonal 645, E-08028 Barcelona Spain; 5 Gabelsbergerstraße 2, 30163 Hannover, Germany Gabelsbergerstraße 2 30163 Hannover Germany; 6 CURCULIO Institute e.V., Hauweg 62, D-41066 Mönchengladbach, Germany CURCULIO Institute e.V., Hauweg 62 D-41066 Mönchengladbach Germany; 7 Department of Life, Health & Environmental Sciences, University of L’Aquila, 67100 L’Aquila, Italy Department of Life, Health & Environmental Sciences, University of L’Aquila 67100 L’Aquila Italy; 8 Centre for Ecology, Evolution and Environmental Changes and Azorean Biodiversity Group, Faculty of Sciences and Technology, University of the Azores, Ponta Delgada, Azores, Portugal Centre for Ecology, Evolution and Environmental Changes and Azorean Biodiversity Group, Faculty of Sciences and Technology, University of the Azores Ponta Delgada, Azores Portugal

**Keywords:** Arthropoda, Azores, Terceira Island, coastal area, standardised sampling

## Abstract

**Background:**

During a LIFE research project aiming at the implementation of the conservation of the habitats and restoration of coastal wetland areas of Praia da Vitória (Terceira, Azores, Portugal), there was the opportunity undertake a systematic record of several groups of arthropods in three wetland areas: Paul da Praia da Vitória (PPV), Paul do Belo Jardim (PBJ) and Paul da Pedreira do Cabo da Praia (PPCP). The objective of the study was to perform a rapid biodiversity assessment, comparing the three sites in two different years, before and after the implementation of several conservation measures. This project also contributed to improve the knowledge of Azorean arthropod diversity at both local and regional scales, including new taxa for Terceira island and new records for Azores. Taking into consideration those aims, a set of standardised sampling methods were performed, inspired by the COBRA protocol originally developed for spiders.

**New information:**

A total of 15,810 specimens belonging to 216 arthropod species and subspecies were collected. Beetles (Insecta, Coleoptera) and spiders (Araneae) dominated, with 81 and 51 taxa, respectively. Two beetle families dominated, Staphylinidae and Curculionidae with, respectively, 22 and 17 species and subspecies. Exotic species were also dominant (132 species and subspecies), the Azorean endemics being restricted to only eight taxa. The remaining 76 species and subspecies are native non-endemic. Two rare endemic species were found with relatively sustainable populations, the Azores Cone-head *Conocephalus chavesi* (Orthoptera, Tettigoniidae) and the true weevil *Drouetius oceanicus oceanicus* (Coleoptera, Curculionidae). A total of six species are novel for the Azores, five exotic (*Bledius unicornis, Carpelimus zealandicus, Oenopia doublieri, Sitona hispidulus, Trichiusa immigrata*) and one possibly native (*Pyrrhocoris apterus*). An additional 15 taxa are novel for Terceira island, ten exotic (*Cheiracanthium mildei*, *Cylindroiulus latestriatus*, *Eumodicogryllus bordigalensis, Nemobius sylvestris*, *Pissodes castaneus*, *Psyllipsocus ramburi*, *Trachyzelotes lyonneti*, *Trigonnidium cicindeloides*, *Tychius cuprifer*, *Zelotes tenuis*) and five native (*Aegialia arenaria*, *Oxypoda lurida*, *Platycleis sabulosa*, *Plinthisus brevipennis*, *Tachyura diabrachys*).

## Introduction

The terrestrial coastal lines of the Azores include important wetland areas, namely salty lakes. These habitats were subject to intense human disturbance and, after almost 600 years of human occupancy, only very few coastal wetland habitats still persist in these Atlantic islands. Despite these impacts, three small areas are still available in Terceira Island: i) a native but highly modified coastal saltmarsh habitat, Paul Praia da Vitória (PPV); ii) a new coastal saltmarsh that was created by rehabilitation of the quarry at Cabo da Praia, Paul da Pedreira do Cabo da Praia (PPCP) (Morton et al. 1997); iii) a wetland included in a dune area, the Paul do Belo Jardim (PBJ). The knowledge of the arthropod fauna of these habitats was until recently very incipient, but more recently, the LIFE project "Ecological Restoration and Conservation of Praia da Vitória Coastal Wet Green Infrastructure" (2013-2018) implemented a two-year inventory and monitoring of the biota in these wetland areas. As a consequence, a first survey was conducted in 2016 in order to compare the diversity of arthropods in ground and aerial habitats (herbaceous, shrubs and trees) in the referred wetland areas (Borges et al. 2017). A second survey was performed in 2017, repeating the same sampling protocols with some additional sampling.

## General description

### Purpose

In this contribution, we present detailed data on the distribution and abundance of species belonging to several groups of arthropods in three Terceira Island (Azores) wetlands during two years (2016-2017). In addition, we list the new taxonomic records for the Azores or Terceira Island. In doing this, we are contributing to address two key biodiversity shortfalls (see Cardoso et al. 2011): i) the need for improving current information on the local and regional distribution of Azorean arthropods (the Wallacean shortfall); and ii) the need for collecting abundance data for future monitoring purposes (the Prestonian shortfall).

## Project description

### Title

The inventory of selected groups of terrestrial arthropods in three coastal wetlands from Terceira Island (Azores)

### Personnel

The inventory was conducted during two years (2016-2017) under the responsibility of Paulo A. V. Borges with constant participation of César Pimentel. For the night sampling, additional help in the field was provided by Rosalina Gabriel and Mariana Brito. A large group of taxonomists contributed for the species identification: Luís Crespo (Araneae); Artur Serrano (Insecta, Coleoptera); Volker Assing and Michael Schülke (Coleoptera, Staphylinidae); António O. Soares (Coleoptera, Coccinellidae); Simone Fattorini (Coleoptera, Tenebrionidae); Peter Stüben (Coleoptera, Curculionidae). Finally, in the lab, we had the support of Alejandra Ros-Prieto in vouchers management for the University of Azores Insect Collection "Dalberto Teixeira Pombo" and Enésima Mendonça for the database management.

### Study area description

Terceira Island (area: 400.6 km²; elevation: 1,021.14 m) is one of the nine islands from the Azores archipelago, located in the North Atlantic, roughly at 38°43′49″N 27°19′10″W. The climate in the Azores is temperate oceanic, with regular and abundant rainfall, with high levels of relative humidity and persistent winds, mainly during the winter and autumn seasons.

**Figure 1.**
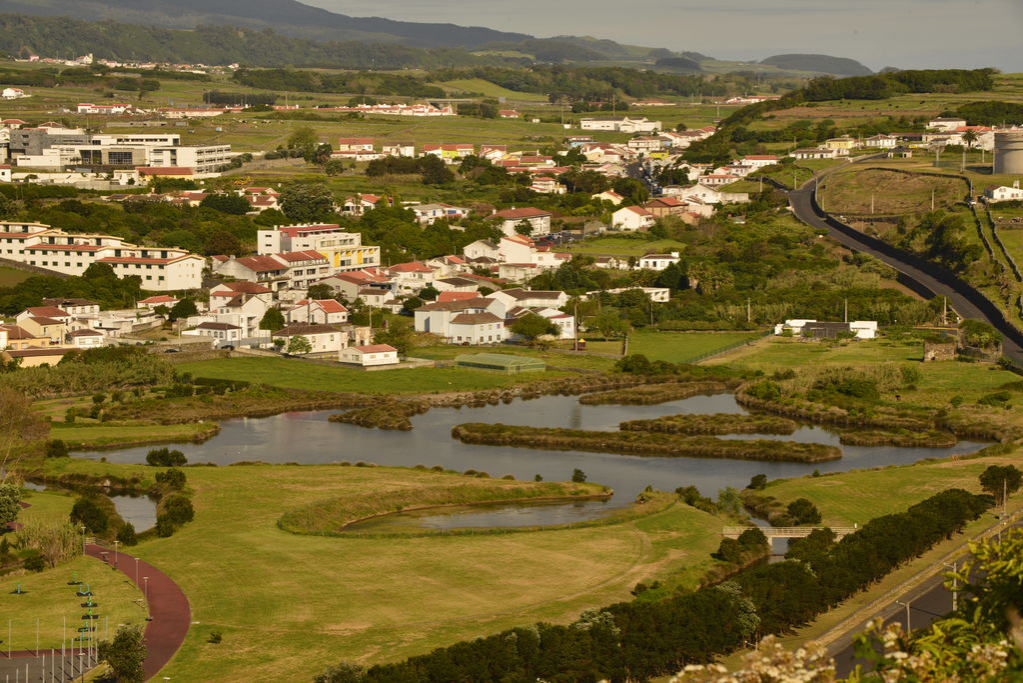
General aspect of Paul da Praia da Vitória with its islands and the surrounding urban area (Photo by Paulo A.V. Borges).

**Figure 2.**
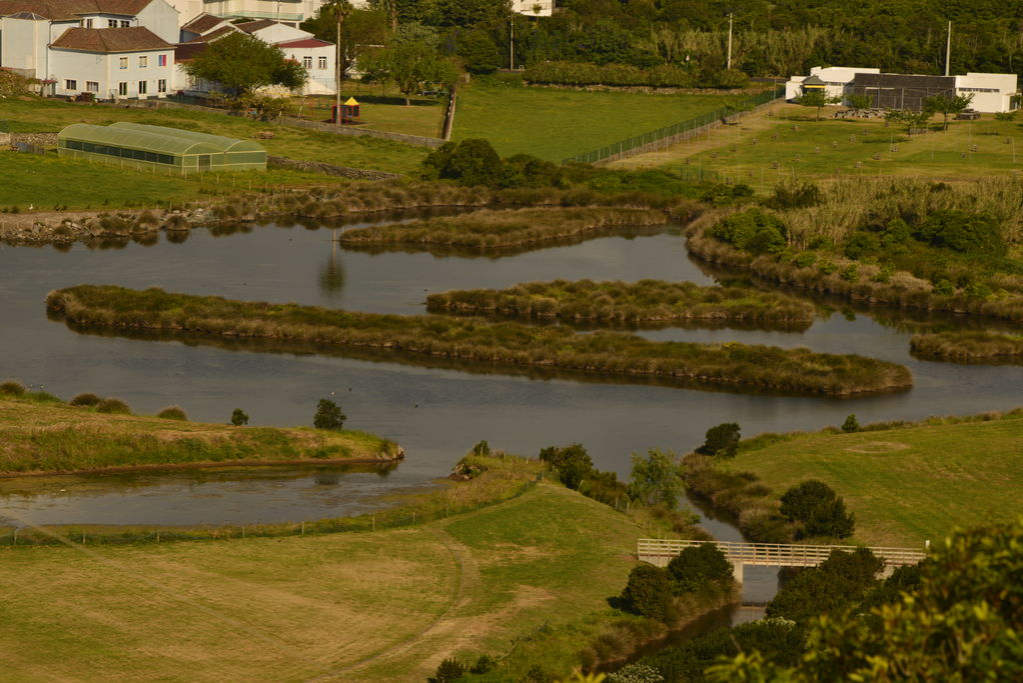
Detail of the recently created islands of *Juncus acutus* habitat in Paul da Praia da Vitória (Photo by Paulo A.V. Borges).

Terceira Island is known for the presence of some very important pristine areas at high elevation (Gaspar et al. 2011). However, few natural areas still remain at lower elevations, notably in Praia da Vitória county. Three wetland areas, Paul da Praia da Vitória (PPV), Paul do Belo Jardim (PBJ) and Paul da Pedreira do Cabo da Praia (PPCP) (Figs 1, 2) were studied in this project. Coastal vegetation dominates, namely *Juncus acutus* and still includes some arboreal cover by the native shrub *Morella faya.* The *Erica*-*Morella* coastal woodlands as described in Elias et al. (2016) are not present and the exotic invasive species *Arundo donax* is very common.

**Figure 3.**
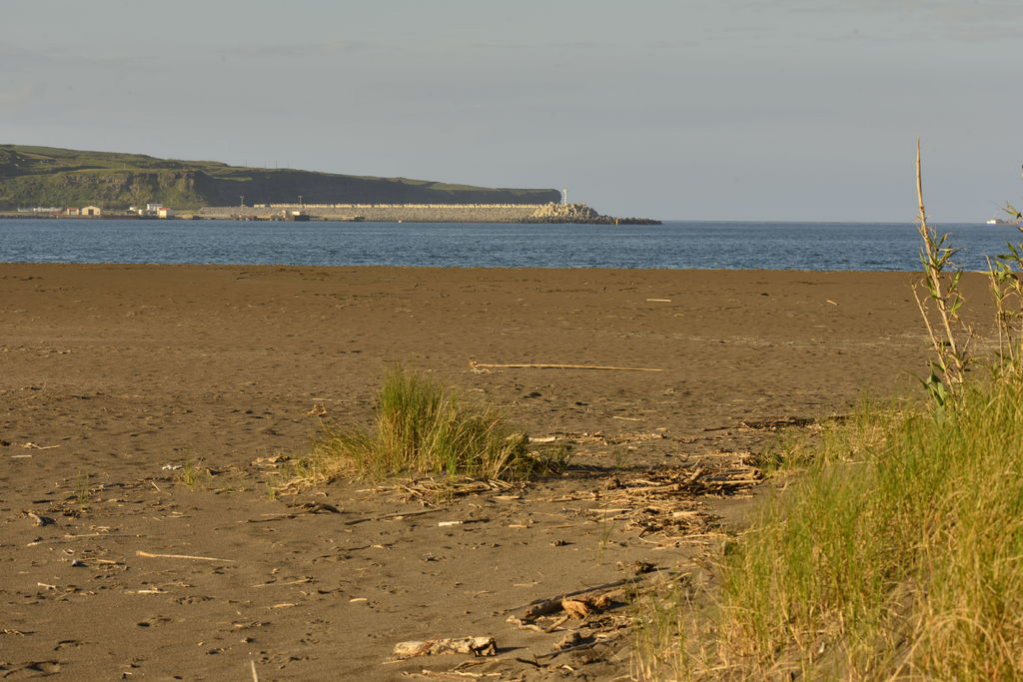
Paul do Belo Jardim dune area (Photo by Paulo A.V. Borges).

**Figure 4.**
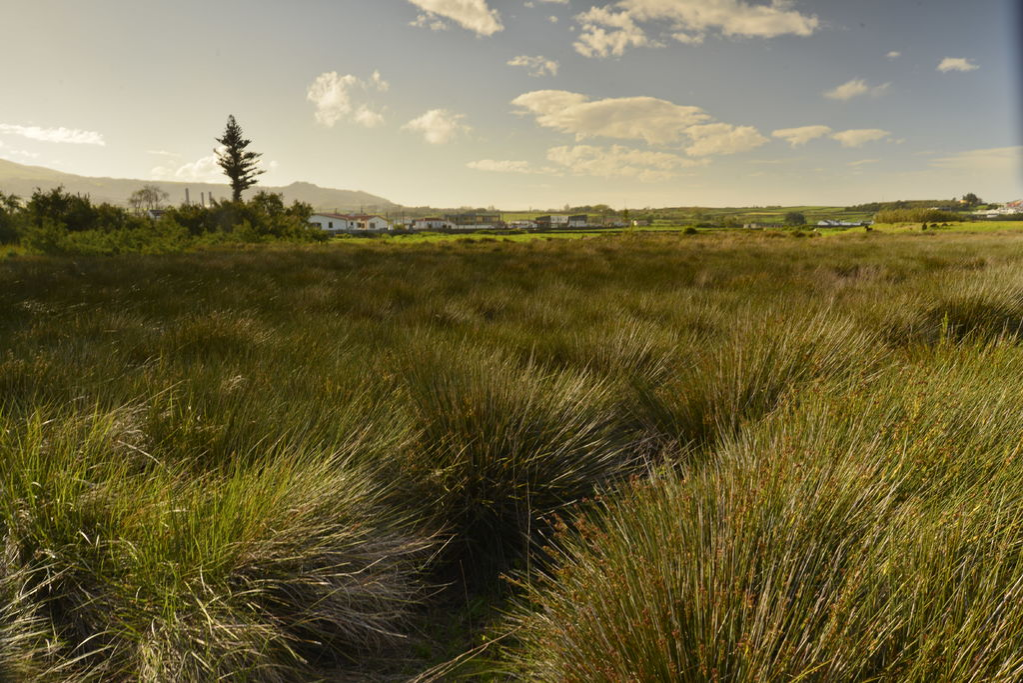
Paul do Belo Jardim *Juncus effusus* area (Photo by Paulo A.V. Borges).

**Figure 5.**
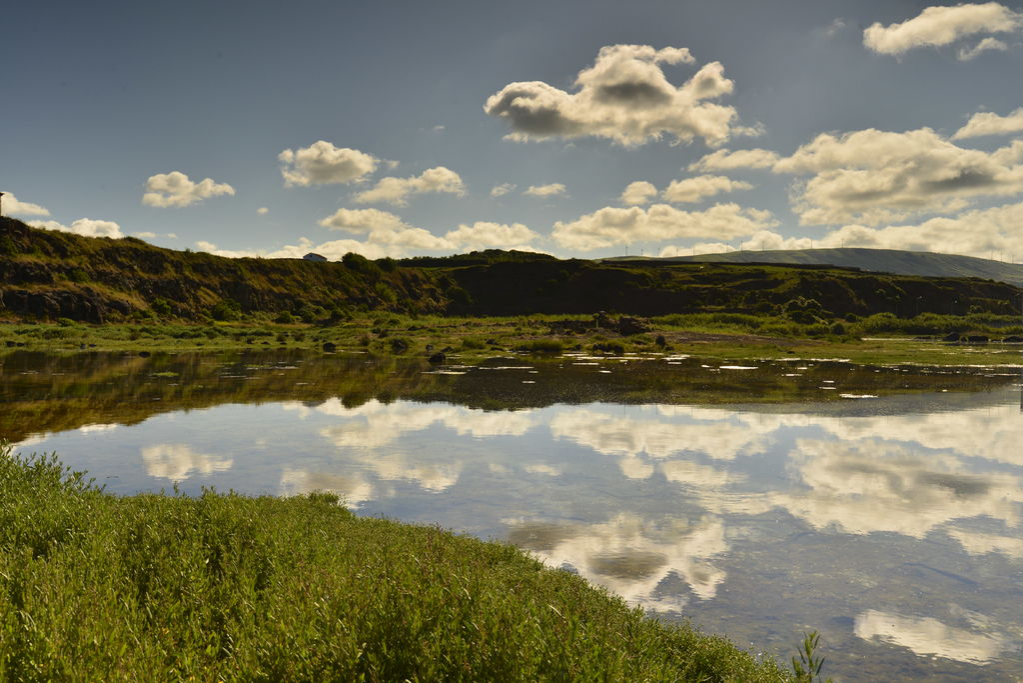
Paul da Pedreira do Cabo da Praia (Photo by Paulo A.V. Borges).

**Figure 6.**
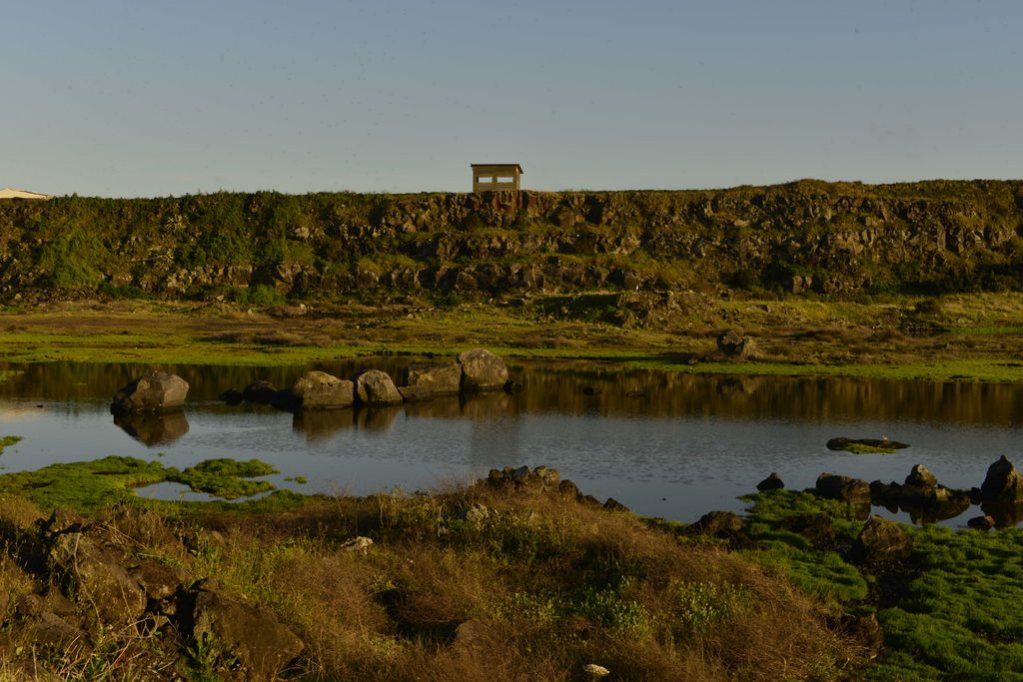
Paul da Pedreira do Cabo da Praia detail of margins (Photo by Paulo A.V. Borges).

The PPV (Fig. 1) was a large coastal salty marshland with associated dunes, which was largely transformed and reduced for urban development and underwent several dynamic changes in the last 500 years of human occupation. After some major work performed between 2006 and 2010, PPV is currently characterised by a large waterbody with islands of *Juncus acutus* isolated by channels (Fig. 2). PBJ was originally one of the largest dune areas from the Azores (Fig. 3), but after the construction of the Praia da Vitória harbour, it was reduced to a very small wetland area, with a dune covered partially by *J. acutus* (Fig. 4). Of particular relevance is the presence of a small stream adding some diversity of vegetation and arthropods (Borges et al. 2017). The case of PPCP is completely different, since this is a recently created wetland (Fig. 5), resulting from the removal of large amounts of stones for the construction of the Praia da Vitória harbour, around 1980 (Fig. 6). As a consequence a new ecosystem was created, the quarry of Cabo da Praia (Morton et al. 1997).

### Design description

In each of the three wetland areas, transects were setup to allow the sampling of epigean arthropods in the main habitats.

In PPV, three main transects were setup: i) PPV-T200 (Paul da Praia Vitória - Margins) that covers the main margins of the water bodies; ii) PPV-T201 (Paul da Praia Vitória - Island) that covers some of the isolated islands; iii) PPV-T205 (Paul da Praia Vitória - Cerrado São Lazaro) to sample an historical locality with a high diversity of ground-beetle species (Borges 1995; Borges et al. 2017).

In PBJ, two transects were setup: i) PBJ-T203 (Paul do Belo Jardim - Margins), which was located within the *Juncus acutus* plants; ii) PBJ-T204 (Paul do Belo Jardim - Stream), which was setup in a small stream.

In PPCP, only one transect was setup, PPCP-T202 (Paul da Pedreira do Cabo da Praia - Margins), which covers the main margins of the water.

The beating and sweeping samples were conducted both during the day and night and were undertaken by walking randomly within the sites.

### Funding

This study was financed by the project LIFE+ (LIFE12 BIO/PT/000110: Ecological Restoration and conservation Infrastructure Green Wet Coast Praia da Vitória) (2013-2018).

## Sampling methods

### Study extent

This study covers a small coastal area with 3.58 km extension between PPV and PPCP.

### Sampling description

In each site, arthropods were sampled during the summers of 2016 and 2017 using a combination of standardised methods inspired by the COBRA protocol (Cardoso 2009):

- Nocturnal active aerial searching (AAS): Four samples were obtained by four trained collectors (Paulo Borges, Mariana Brito, Rosalina Gabriel, César Pimentel) targeting active arthropods found above knee-level by hand, forceps, pooter or brush and immediately transferring them into vials containing alcohol. All the time spent in searching (one hour per researcher) was accounted for.
- Foliage Beating (FB): During daytime, ten samples from each dominant tree or bush were taken. A 110 cm × 80 cm sheet with a frame was used as a drop-cloth (beating tray) and a wooden pole of at least 1.5 m was used to beat tree branches, as high as possible. The plants selected were: *A. donax* and *M. faya* in PPV and PPCP and *A. donax* and *J. acutus* in PBJ. In 2017, in addition, two samples during the night (FSN) were obtained (one hour each sample covering several plants).
- Foliage sweeping (FS): A round sweep net with an opening diameter of 46 cm was used to sweep bushes and tall herbs. All time spent sweeping or searching for dislodged arthropods was accounted for. Two samples during daytime (FSD) were obtained (one hour each sample). In 2017, in addition, two samples during the night (FSN) were obtained (one hour each sample).
- Pitfall (PIT): Pitfall traps (4.2 cm wide at the top and approximately 7.2 cm deep) were placed immediately outside the perimeter of each lake, spaced 10 metres apart. Traps were filled with 3–4 cm of 100% propylene glycol and left in the field for seven days. Traps were protected from predation, inundation with rainwater and unwanted vertebrate capture by using plates sitting on stilts 2 cm above the ground surface. In PBJ, two transects were performed with 30 traps in the main transect and 15 traps in a secondary transect covering a small stream. In PPV and PPCP, single transects of 30 traps each were setup in the margins of water bodies. In PPV, half of the traps were in the margins of the largest “island”. In 2017, additional traps were setup in Cerrado São Lazaro (PPV-T205 Paul da Praia Vitória).

For each site, a total of four samples of AAS, 20 samples of FB, two samples of FS and 30 main samples of PIT were obtained, totalling 56 samples per site and an overall 168 samples in 2017. Further, in 2017, additional pitfall traps in the PBJ small stream added 15 more samples totalling 183 samples. The main 56 samples per site included the sampling of two main sub-habitats, the aerial vegetation with 26 samples (20 beatings during the day, two sweeps during the day and four night aerial searches) and the ground habitat with 30 pitfall samples.

In 2017, the additional samples made during the night added four samples for each site, totalling 60 samples per site. Accumulation curves were performed and completeness was high for all sites (see Borges et al. 2017).

### Quality control

The correct identification of the sampled taxa is crucial. We followed a three-step process to identify arthropod species: (1) for arthropod orders for which there was taxonomic expertise, one of us (CP) performed morphospecies sorting using a parataxonomy approach (see Oliver and Beattie 1993) with a reference collection; (2) a trained taxonomist (PAVB) corrected all the splitting and lumping errors and identified most of the species; and 3) the morphospecies for which a correct identification was not possible were sent to experts for identification. Taxonomic nomenclature followed the arthropod checklist in Borges et al. (2010) and for the new six taxa the following taxonomic references were used: Lohse 1984, Quinn and Hower 1986, Smaili et al. 2009, Schülke and Smetana 2015.

## Geographic coverage

### Description

Terceira Island (Azores), Macaronesia, Portugal

### Coordinates

 and 38°42’47.95’ Latitude; 27°03’40.93’ and Longitude.

## Taxonomic coverage

### Description

Arthropods including Diplopoda, Chilopoda, Arachnida (Opiliones; Pseudoscorpiones; Araneae) and Hexapoda (Microcoryphia; Zygentoma; Odonata; Orthoptera; Phasmatodea; Dermaptera; Psocoptera; Hemiptera; Thysanoptera; Neuroptera; Coleoptera; Lepidoptera; Hymenoptera - Formicidae)

## Temporal coverage

### Notes

The sampling was performed on two occasions: summer 2016 and summer 2017.

## Collection data

### Collection name

Dalberto Teixeira Pombo insect collection at the University of Azores.

### Collection identifier

DTP

### Specimen preservation method

All specimens were preserved in 96% ethanol

### Curatorial unit

Dalberto Teixeira Pombo insect collection at the University of Azores.

## Usage rights

### Use license

Open Data Commons Attribution License

### IP rights notes

Additional information on this study may also be requested to the first author

## Data resources

### Data package title

LIFE_CWR_TER_Arthropods

### Resource link

http://ipt.gbif.pt/ipt/resource?r=arthrop_pv_ter_az

### Alternative identifiers

http://islandlab.uac.pt/software/ver.php?id=30

### Number of data sets

1

### Data set 1.

#### Data set name

Arthropods from Praia da Vitória

#### Data format

Darwin Core Archive

#### Number of columns

60

#### Download URL

http://ipt.gbif.pt/ipt/resource?r=arthrop_pv_ter_az

#### Data format version

version 1

#### Description

In this data table, we include all the records for which a taxonomic identification of the species was possible. The dataset submitted to GBIF is structured as a sample event dataset, with two tables: event (as core) and occurrences. The data in this sampling event resource has been published as a Darwin Core Archive (DwC-A), which is a standardised format for sharing biodiversity data as a set of one or more data tables. The core data table contains 343 records. One extension data table also exists. An extension record supplies extra information about a core record. The number of records in each extension data table is illustrated in the IPT link.

This IPT archives the data and thus serves as the data repository. The data and resource metadata are available for downloading in the downloads section. The versions table lists other versions of the resource that have been made publicly available and allows tracking changes made to the resource over time.

In Suppl. material 1, we provide a simpler dataset with few columns in a single table.

**Data set 1.** 

Column label	Column description
Table Event	The sub-table with events
eventID	Identifier of the events, unique for the dataset
eventDate	Date or date range the record was collected
eventTime	Time of the day the record was collected
samplingProtocol	The sampling protocol used to capture the species
samplingEffort	The amount of time of each sampling
sampleSizeValue	The numeric amount of time spent in each sampling
sampleSizeUnit	The unit of the sample size value
locationID	Identifier of the location
fieldNumber	Number given to each sample
decimalLatitude	Approximate centre point decimal latitude of the field site in GPS coordinates
decimalLongitude	Approximate centre point decimal longitude of the field site in GPS coordinates
geodeticDatum	The reference point for the various coordinate systems used in mapping the earth
coordinatePrecision	Precision of the coordinates
coordinateUncertaintyInMeters	Uncertainty of the coordinates
georeferenceSources	Method used to obtain coordinates
minimumElevationInMetres	Minimum elevation in metres
maximumElevationInMetres	Maximum elevation in metres
country	Country of the sampling site
countryCode	ISO code of the country of the sampling site
stateProvince	Name of the region of the sampling site
islandGroup	Name of archipelago
island	Name of the island
municipality	Name of the municipality
locality	Name of the locality
locationRemarks	Details on the locality site
verbatimCoordinates	Original coordinates recorded
Table Occurrences	The sub-table with occurrence data
type	Type of the record, as defined by the Public Core standard
modified	Date of the last modification of the record
eventID	Identifier of the events, unique for the dataset
licence	Reference to the licence under which the record is published
occurrenceID	Identifier of the record, coded as a global unique identifier
basisOfRecord	The nature of the data record
InstitutionID	The identity of the institution publishing the data
InstitutionCode	The code of the institution publishing the data
collectionCode	The code of the collection where the specimens are conserved
datasetName	Name of the dataset
catalogNumber	Record number of the specimen in the collection
recordedBy	Name of the person who performed the sampling of the specimens
identifiedBy	Name of the person who made the identification
dateIdentified	Date on which the record was identified
scientificName	Complete scientific name including author and year
taxonRank	Lowest taxonomic rank of the record
kingdom	Kingdom name
phylum	Phylum name
class	Class name
order	Order name
family	Family name
genus	Genus name
specificEpithet	Specific epithet
infraspecificEpithet	Infraspecific epithet, when available
individualCount	Total number of individuals captured
organismQuantity	Total number of individuals captured, as numeric
organismQuantityType	The unit of the identification of the organisms
sex	The sex and quantity of the individuals captured
lifeStage	The life stage of the organisms captured
scientificNameAuthorship	Name of the author of the lowest taxon rank included in the record
establishmentMeans	The process of establishment of the species in the location, using a controlled vocabulary: 'native non-endemic', 'introduced', 'endemic'
occurrenceRemarks	Remarks on the occurrence with the plant species from where the specimens where captured

## Additional information

We collected and identified 15,810 specimens representing 216 species or subspecies and 197 genera during this study (Table 1). Beetles (Insecta, Coleoptera) and spiders (Araneae) were the most diverse taxa, with 81 and 51 taxa, respectively. Two beetle families were also diverse, Staphylinidae and Curculionidae with, respectively, 22 and 17 species and subspecies. Exotic species dominated with 132 species and subspecies, the Azorean endemics being restricted to only eight taxa. The remaining 76 species and subspecies are native non-endemic.

The most abundant species, belonging to the first quartile when ranking species abundances, accounted for 14,680 specimens, i.e. 93% of all adult sampled specimens belong to 25% of the species (54 species). From these 54 species, four are endemic, 22 are native and 28 are exotic. Thirty one species had more than 100 specimens and four of them were particularly abundant: the native ant *Lasius grandis* with 3140 specimens, the native harvestman *Leiobunum blackwalli* (Opiliones) with 1090 individuals, the native beetle *Stilbus testaceus* with 956 specimens and the native ant *Monomorium carbonarium* with 776 individuals.

Only one of the three most abundant ground-beetles recorded for PPV in 1991-1993 (Borges 1995) was found in the current sample, but with low abundance: *Bembidion semipunctatum*. The species was found in PPV (with 27 specimens), but also in PPCP with only two specimens

**Figure 7.**
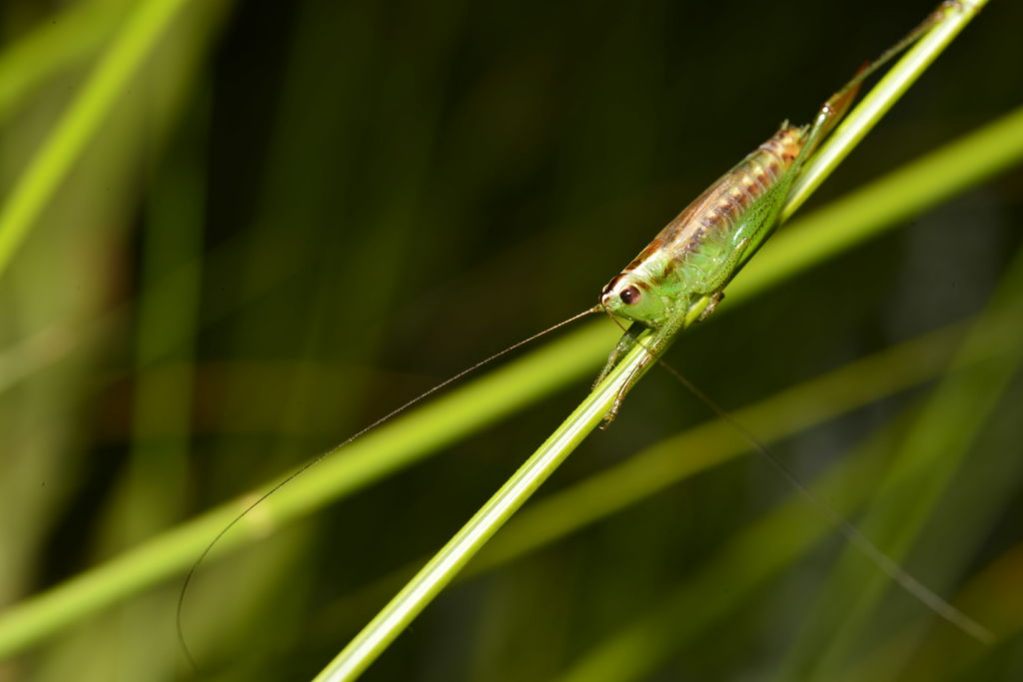
A juvenile of Azores Cone-head *Conocephalus chavesi* (Orthoptera, Tettigoniidae) (Photo by Paulo A.V. Borges).

**Figure 8.**
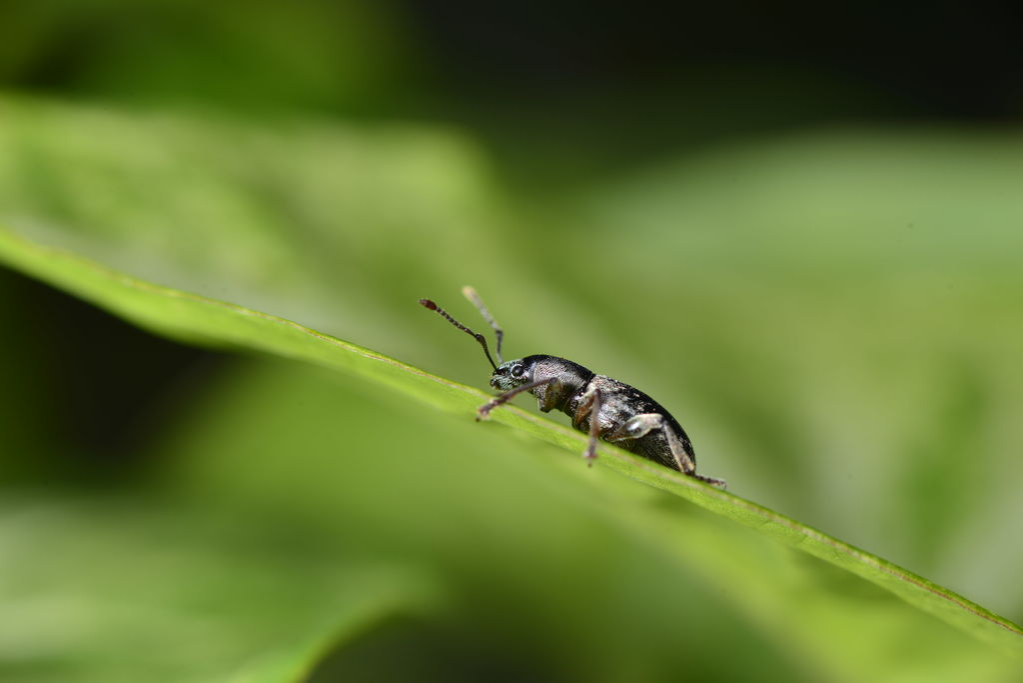
The Azores endemic true weevil *Drouetius oceanicus oceanicus* (Coleoptera, Curculionidae) (Photo by Paulo A.V. Borges).

Paul Belo Jardim (PBJ) was the richest site with 148 species and subspecies, the other two sites having equal diversity (Table 1). Particularly relevant was the finding of two rare endemic species in PBJ, the Azores Cone-head *Conocephalus chavesi* (Orthoptera, Tettigoniidae) (Fig. 7), that was recently listed as Endangered by IUCN (Hochkirch and Borges 2016) and the true weevil *Drouetius oceanicus oceanicus* (Coleoptera, Curculionidae) (Fig. 8), that was recently listed as Endangered by IUCN (see Borges and Lamelas-López 2018). The Azores Cone-head *Conocephalus chavesi* was also found in the two other sites but with lower abundance.

### Known ranges and ecology of newly reported species

Twenty-one species, which represent 10% of the total species collected, are new records for either the Azores and Terceira island (six species) or only Terceira Island (15 species). The new species for the Azores include five exotic and one possibly native species. The 15 new records for Terceira island include ten exotic and five native species (see also Table 1).

**Diplopoda - Julida**

- *Cylindroiulus latestriatus* (Curtis, 1845) (new for Terceira island). Previously recorded on five islands (Corvo, Flores, Faial, S. Miguel and S. Maria). Exotic species common in Western Europe. This species is usually associated with coastal and dune systems (Kime 2004). Captured with pitfall traps.

**Araneae**

- *Cheiracanthium mildei* L. Koch, 1864 (new for Terceira island). Previously recorded on two islands (Flores and S. Miguel). This is an exotic spider native from Europe, North Africa, Turkey and the Near East. Introduced to North America, Argentina and Azores. (see World Spider Catalog 2018). The species was found mostly in the canopy of *Morella faya*.

- *Trachyzelotes lyonneti* (Audouin, 1926) (new for Terceira island). Previously recorded on four islands (Faial, Graciosa, S. Miguel and S. Maria). This is an exotic spider native from the Mediterranean to Central Asia. The species has been introduced into the United States, Mexico, Peru and Brazil (see World Spider Catalog 2018). Captured with pitfall traps.

- *Zelotes tenuis* (L. Koch, 1866) (new for Terceira island). Previously recorded on a single island (S. Miguel). This is an exotic spider, native from the Mediterranean to Russia (Caucasus). Introduced to Galapagos Is., Azores and USA (see World Spider Catalog 2018). Captured with pitfall traps.

**Insecta - Orthoptera**

- *Eumodicogryllus bordigalensis* (Latreille, 1804) (new for Terceira island). Previously recorded on two islands (S. Miguel and S. Maria). This is an exotic species native from N-Africa, S-Europe and warmer parts of Asia. It is spreading northwards due to climate change. It has already reached southern parts of West Germany (see Anonymous 2018a). Captured with pitfall traps.

- *Nemobius sylvestris* (Bosc D’Antic, 1792) (new for Terceira island). Previously recorded on a single island (S. Miguel). This is an exotic species, native from North Africa across the Iberian Peninsula, France, north-westernmost Italy and parts of Central Europe to southern England, south-western Poland and the Czech Republic (see Anonymous 2018c). Captured with pitfall traps.

- *Platycleis sabulosa* Azam, 1901 (new for Terceira island). This is a possible native species with origin in Northern Africa and South-western Europe (Iberian Peninsula, Southern France) (see Anonymous 2018b). Captured with pitfall traps.

- *Trigonnidium cicindeloides* Rambur, 1839 (new for Terceira island). First recorded for Azores (S. Miguel) by Borges et al. (2013) and now also found in Terceira. This is a southern Europe (Mediterranean area) native species, but occurs also on the Canary Islands, Africa, Madagascar, China, Japan and Korea. This species if frequently found associated with ponds. Captured with pitfall traps.

**Insecta - Hemiptera**

- *Plinthisus brevipennis* (Latreille, 1807) (new for Terceira island). Previously recorded on five islands (Faial, Pico, Graciosa, S. Miguel and S. Maria). This is a native species usually associated with grassy environments. Captured with pitfall traps.

- *Pyrrhocoris apterus* (Linnaeus,1758) (new for the Azores). This is a very common and widespread Palaearctic species. This is possibly a native species from Azores. Captured with pitfall traps, but also associated with *Arundo donax*.

**Insecta - Psocoptera**

- *Psyllipsocus ramburi* Sélys-Longchamps, 1872 (new for Terceira island). Previously recorded on two islands (S. Miguel and S. Maria). This is an exotic species in Azores and native from West Palaearctic. Captured with pitfall traps, this species is usually associated with damp sites (Robinson 2005).

**Insecta - Coleoptera**

- *Aegialia arenaria* (Fabricius, 1787) (new for Terceira island). Previously recorded on a single island (S. Miguel). This is a native dune scarab beetle species in Azores and native from West Palaearctic. Captured with pitfall traps, this species is commonly associated with coastal dune areas.

- *Bledius unicornis* (Germar, 1825) (new for the Azores). This is a common rove-beetle species distributed from the Atlantic Islands across Europe and the Mediterranean eastwards to Middle Asia (Schülke and Smetana 2015). Captured with pitfall traps, this species is adapted to damp areas, particularly salt-marsh areas (Zanella and Scarton 2017).

- *Carpelimus zealandicus* (Sharp, 1900) (new for the Azores). Originally most likely from the Australian Region, this species is adventive in Europe, with confirmed records from Central Europe, the British Isles and Scandinavia (Schülke and Smetana 2015). Captured with pitfall traps.

- *Oenopia doublieri* (Mulsant, 1846) (new for the Azores). This exotic species is native from the Mediterranean region. The species was recently recorded also in Morocco and associated with citrus orchards (Smaili et al. 2009). This is, possibly, a recent introduction in the Azores. The species was found associated with the invasive *Arundo donax*.

- *Oxypoda lurida* Wollaston, 1857 (new for Terceira island). Previously recorded on a single island (S. Maria). *Oxypoda lurida* is a widespread and mostly parthenogenetic species distributed from the Atlantic Islands across Europe and the Mediterranean eastwards to Turkey and Cyprus) (Schülke and Smetana 2015). Captured with pitfall traps.

- *Pissodes castaneus* (De Geer, 1775) (new for Terceira island). Previously recorded on four islands (Faial, Pico, S. Miguel and S. Maria). The small banded pine weevil is a cosmopolitan species commonly associated with pines, the larval stage having some impact on adult trees. This species is considered invasive (Pestaña and Santolamazza-Carbone 2010) and is widespread on all Macaronesian islands (Stüben 2018) where pines from Europe (e.g. *Pinus sylvestris*) were introduced. Captured with pitfall traps.

- *Sitona hispidulus* (Fabricius, 1777) (new for the the Azores). Known as Clover Root Curculio, this species is native to and widespread throughout Eurasia, but also introduced in North America (Quinn and Hower 1986). Captured with pitfall traps. This species has a short-winged and a long-winged form and prefers stands of *Trifolium* (especially *T. repens*) on damp and relatively dry localities and with a minor preference also for *Medicago* and *Vicia*. It seems to have just arrived into the Azores, otherwise this *Sitona* species could/should have been found even before.

- *Tachyura diabrachys* (Kolenati, 1845) (new for Terceira island). Previously recorded on a single island (S. Maria). This is a west European species. Captured with pitfall traps, this is a species usually associated with damp areas.

- *Trichiusa immigrata* Lohse, 1984 (new for the Azores; Note: there is a mention of this species in the latest edition of the Palaearctic Catalogue, but we have no idea who published the primary record). Originally from North America, this adventive rove-beetle species was first recorded from Central Europe by *Lohse (1984)* and is now widespread and common in the West Palaearctic region from the Atlantic Islands eastwards to Russia and Ukraine. It is usually found in decomposing plant material and in the leaf litter (Moore 2004). The material from the Azores was found in grassland.

- *Tychius cuprifer* (Panzer, 1799) (new for Terceira island). Previously recorded on a single island (S. Miguel). It is also reported from Madeira in 2015 for the first time, collected in multifunnel traps (Stüben 2018). It is most probably introduced with Fabaceae (forage). *T. cuprifer* is a xerothermophilous species from South Europe and North Africa (uninterruptedly until Turkmenistan) and develops mainly on *Trifolium arvense* (it is also called *T. pratense* and *T. stellatum* (CURCULIO_Team 2010).

## Supplementary Material

Supplementary material 1 LIFE_CWR_TER_Arthropods Data type: Occurrences and abundances
Brief description: In this contribution, we present detailed data on the distribution and abundance of species belonging to several groups of arthropods in three Terceira island (Azores) wetlands during two years (2016-2017).
File: oo_217403.XLSX Borges, PAV et al.

## Figures and Tables

**Table 1.** Species abundance per site. PPV – Paul da Praia da Vitória; PBJ – Paul Belo Jardim; PPCP – Paul da Pedreira do Cabo da Praia. END - endemic species from Azores; NAT - native non-endemic species; INTR - exotic species.

Class	Order	Taxon	Colonization	PPV	PBJ	PPCP
Arachnida	Araneae	*Altella lucida*	INTR	1		
Arachnida	Araneae	*Arctosa perita*	INTR	1	84	1
Arachnida	Araneae	*Argiope bruennichi*	NAT	23	154	6
Arachnida	Araneae	*Cheiracanthium mildei*	INTR	76	18	1
Arachnida	Araneae	*Clubiona decora*	NAT	191	57	86
Arachnida	Araneae	*Clubiona terrestris*	INTR	30		
Arachnida	Araneae	*Cryptachaea blattea*	INTR	6		
Arachnida	Araneae	*Dysdera crocata*	INTR	12	47	12
Arachnida	Araneae	*Eidmannella pallida*	INTR	1		
Arachnida	Araneae	*Emblyna acoreensis*	END	144	47	191
Arachnida	Araneae	*Entelecara schmitzi*	INTR	1	9	7
Arachnida	Araneae	*Erigone autumnalis*	INTR	5	9	
Arachnida	Araneae	*Erigone dentipalpis*	INTR	3	6	9
Arachnida	Araneae	*Ero aphana*	INTR			1
Arachnida	Araneae	*Ero furcata*	INTR	1	6	
Arachnida	Araneae	*Heliophanus kochii*	INTR		3	6
Arachnida	Araneae	*Macaroeris cata*	NAT	4	4	
Arachnida	Araneae	*Macaroeris diligens*	NAT	127	45	120
Arachnida	Araneae	*Malthonica pagana*	INTR		1	
Arachnida	Araneae	*Mangora acalypha*	INTR			1
Arachnida	Araneae	*Mermessus bryantae*	INTR		1	
Arachnida	Araneae	*Mermessus fradeorum*	INTR	2	1	1
Arachnida	Araneae	*Metellina merianae*	INTR		6	8
Arachnida	Araneae	*Neoscona crucifera*	INTR	26	12	25
Arachnida	Araneae	*Neottiura bimaculata*	INTR		1	
Arachnida	Araneae	*Nigma puella*	INTR	3		88
Arachnida	Araneae	*Oecobius navus*	INTR		1	
Arachnida	Araneae	*Oedothorax fuscus*	INTR	91	115	205
Arachnida	Araneae	*Ostearius melanopygius*	INTR		4	3
Arachnida	Araneae	*Pachygnatha degeeri*	INTR	6	9	2
Arachnida	Araneae	*Parasteatoda tepidariorum*	INTR	4		1
Arachnida	Araneae	*Pardosa acorensis*	END		9	50
Arachnida	Araneae	*Pelecopsis parallela*	INTR		4	
Arachnida	Araneae	*Phidippus audax*	INTR	47	104	3
Arachnida	Araneae	*Prinerigone vagans*	INTR			1
Arachnida	Araneae	*Pseudeuophrys vafra*	INTR	3		
Arachnida	Araneae	*Salticus mutabilis*	INTR	3	4	10
Arachnida	Araneae	*Segestria florentina*	INTR			4
Arachnida	Araneae	*Steatoda grossa*	INTR	7	3	
Arachnida	Araneae	*Steatoda nobilis*	INTR	4	4	4
Arachnida	Araneae	*Synageles venator*	INTR	22	11	14
Arachnida	Araneae	*Tegenaria domestica*	INTR	2	3	6
Arachnida	Araneae	*Tenuiphantes tenuis*	INTR	43	34	2
Arachnida	Araneae	*Tetragnatha extensa*	INTR	39	3	6
Arachnida	Araneae	*Theridion hannoniae*	INTR			1
Arachnida	Araneae	*Theridion melanostictum*	INTR	4	7	6
Arachnida	Araneae	*Theridion musivivum*	NAT		2	
Arachnida	Araneae	*Trachyzelotes lyonneti*	INTR		1	1
Arachnida	Araneae	*Xysticus nubilus*	INTR	24	218	164
Arachnida	Araneae	*Zelotes aeneus*	INTR	17	11	16
Arachnida	Araneae	*Zelotes tenuis*	INTR		6	
Arachnida	Araneae	*Zodarion atlanticum*	INTR			1
Arachnida	Araneae	*Zoropsis spinimana*	INTR	5		
Arachnida	Araneae	*Zygiella x-notata*	INTR	6	6	14
Arachnida	Opiliones	*Homalenotus coriaceus*	NAT	47	149	1
Arachnida	Opiliones	*Leiobunum blackwalli*	NAT	157	923	10
Arachnida	Pseudoscorpiones	*Chthonius tetrachelatus*	INTR		2	
Chilopoda	Lithobiomorpha	*Lithobius pilicornis pilicornis*	NAT		13	
Chilopoda	Scutigeromorpha	*Scutigera coleoptrata*	INTR	1	6	14
Diplopoda	Julida	*Choneiulus palmatus*	INTR		2	
Diplopoda	Julida	*Cylindroiulus latestriatus*	INTR	2		
Diplopoda	Julida	*Ommatoiulus moreletii*	INTR	147	510	35
Diplopoda	Julida	*Proteroiulus fuscus*	INTR	1	2	
Diplopoda	Polydesmida	*Oxidus gracilis*	INTR	2	3	
Diplopoda	Polydesmida	*Polydesmus coriaceus*	INTR	63	7	
Insecta	Coleoptera	*Acupalpus flavicollis*	NAT		1	
Insecta	Coleoptera	*Aegialia arenaria*	NAT		1	
Insecta	Coleoptera	*Aeolus melliculus moreleti*	INTR	4		
Insecta	Coleoptera	*Ahasverus advena*	INTR	2		
Insecta	Coleoptera	*Aleochara bipustulata*	INTR		1	
Insecta	Coleoptera	*Amischa analis*	INTR		1	
Insecta	Coleoptera	*Amischa forcipata*	INTR		1	1
Insecta	Coleoptera	*Anisodactylus binotatus*	INTR	2	13	
Insecta	Coleoptera	*Anotylus nitidifrons*	INTR	76	2	
Insecta	Coleoptera	*Aspidapion radiolus*	NAT	3	14	104
Insecta	Coleoptera	*Astenus lyonessius*	NAT		2	
Insecta	Coleoptera	*Atheta fungi*	INTR	4	6	3
Insecta	Coleoptera	*Bembidion semipunctatum*	NAT	27		2
Insecta	Coleoptera	*Bledius unicornis*	INTR			13
Insecta	Coleoptera	*Bradycellus distinctus*	INTR		1	
Insecta	Coleoptera	*Calymmaderus solidus*	INTR		1	
Insecta	Coleoptera	*Carpelimus corticinus*	NAT	2		
Insecta	Coleoptera	*Carpelimus zealandicus*	INTR	1		
Insecta	Coleoptera	*Cartodere bifasciata*	INTR		1	
Insecta	Coleoptera	*Cercyon haemorrhoidalis*	INTR	1		3
Insecta	Coleoptera	*Chrysolina bankii*	NAT	11		1
Insecta	Coleoptera	*Coccinella undecimpunctata undecimpunctata*	INTR		11	
Insecta	Coleoptera	*Coccotrypes carpophagus*	INTR	1		
Insecta	Coleoptera	*Coelositona puberulus*	INTR			3
Insecta	Coleoptera	*Cordalia obscura*	INTR	15	38	5
Insecta	Coleoptera	*Creophilus maxillosus maxillosus*	INTR		4	
Insecta	Coleoptera	*Cryptamorpha desjardinsii*	INTR	21	8	7
Insecta	Coleoptera	*Drouetius oceanicus oceanicus*	END		5	
Insecta	Coleoptera	*Enochrus bicolor*	INTR	5		1
Insecta	Coleoptera	*Epitrix hirtipennis*	INTR		2	
Insecta	Coleoptera	*Gonipterus scutellatus*	INTR	1		
Insecta	Coleoptera	*Gymnetron pascuorum*	INTR	1	3	2
Insecta	Coleoptera	*Heteroderes azoricus*	END	13	12	10
Insecta	Coleoptera	*Heteroderes vagus*	INTR	20	219	11
Insecta	Coleoptera	*Hirticollis quadriguttatus*	NAT	32	92	
Insecta	Coleoptera	*Hypera postica*	INTR			1
Insecta	Coleoptera	*Hypocaccus brasiliensis*	INTR		21	
Insecta	Coleoptera	*Kalcapion semivittatum semivittatum*	NAT			3
Insecta	Coleoptera	*Laemostenes complanatus*	INTR			2
Insecta	Coleoptera	*Lixus pulverulentus*	INTR	1		
Insecta	Coleoptera	*Meligethes aeneus*	INTR		3	6
Insecta	Coleoptera	*Naupactus leucoloma*	INTR	22	30	53
Insecta	Coleoptera	*Ocypus olens*	NAT		1	
Insecta	Coleoptera	*Oenopia doublieri*	INTR	2	3	1
Insecta	Coleoptera	*Orthochaetes insignis*	NAT	1		
Insecta	Coleoptera	*Otiorhynchus cribricollis*	INTR	24	19	68
Insecta	Coleoptera	*Oxypoda lurida*	NAT			1
Insecta	Coleoptera	*Pantomorus cervinus*	INTR	59	70	3
Insecta	Coleoptera	*Phaleria bimaculata*	INTR		677	
Insecta	Coleoptera	*Phloeonomus punctipennis*	NAT			6
Insecta	Coleoptera	*Phloeostiba azorica*	END	1		
Insecta	Coleoptera	*Pissodes castaneus*	INTR			7
Insecta	Coleoptera	*Platystethus nitens*	NAT		2	1
Insecta	Coleoptera	*Pseudoophonus rufipes*	INTR		5	
Insecta	Coleoptera	*Psylliodes marcidus*	NAT	1		
Insecta	Coleoptera	*Ptenidium pusillum*	INTR	1	6	1
Insecta	Coleoptera	*Pterostichus vernalis*	INTR	1		
Insecta	Coleoptera	*Rhyzobius litura*	NAT			1
Insecta	Coleoptera	*Rodolia cardinalis*	INTR	4		4
Insecta	Coleoptera	*Rugilus orbiculatus orbiculatus*	NAT	1	0	1
Insecta	Coleoptera	*Scymnus interruptus*	NAT	14	14	37
Insecta	Coleoptera	*Scymnus nubilus*	NAT	14	14	37
Insecta	Coleoptera	*Sepedophilus lusitanicus*	NAT		1	
Insecta	Coleoptera	*Sericoderus lateralis*	INTR	11	9	1
Insecta	Coleoptera	*Sitona discoideus*	INTR		3	7
Insecta	Coleoptera	*Sitona hispidulus*	INTR	2		
Insecta	Coleoptera	*Sitona lineatus*	INTR	2		
Insecta	Coleoptera	*Sphenophorus abbreviatus*	INTR		1	
Insecta	Coleoptera	*Stegobium paniceum*	INTR		1	
Insecta	Coleoptera	*Stenolophus teutonus*	NAT	1	2	
Insecta	Coleoptera	*Stethorus pusillus*	NAT			1
Insecta	Coleoptera	*Stilbus testaceus*	NAT	124	175	657
Insecta	Coleoptera	*Tachyporus chrysomelinus*	INTR			1
Insecta	Coleoptera	*Tachyporus nitidulus*	INTR	1	1	
Insecta	Coleoptera	*Tachyura diabrachys*	NAT	1		
Insecta	Coleoptera	*Tribolium castaneum*	INTR		1	
Insecta	Coleoptera	*Trichiusa immigrata*	INTR		1	
Insecta	Coleoptera	*Tychius cuprifer*	INTR		10	7
Insecta	Coleoptera	*Tychius picirostris*	INTR			8
Insecta	Coleoptera	*Typhaea stercorea*	INTR	2	2	1
Insecta	Coleoptera	*Xantholinus longiventris*	INTR		1	
Insecta	Dermaptera	*Euborellia annulipes*	INTR	307	96	120
Insecta	Dermaptera	*Forficula auricularia*	INTR	1	16	14
Insecta	Dermaptera	*Labidura riparia*	NAT		46	38
Insecta	Hemiptera	*Anoscopus albifrons*	NAT	3	3	2
Insecta	Hemiptera	*Beosus maritimus*	NAT	1		
Insecta	Hemiptera	*Buchananiella continua*	INTR	2		18
Insecta	Hemiptera	*Closterotomus norwegicus*	NAT			1
Insecta	Hemiptera	*Cyphopterum adcendens*	NAT	1		
Insecta	Hemiptera	*Emblethis denticollis*	NAT		1	
Insecta	Hemiptera	*Empicoris rubromaculatus*	INTR	3	3	1
Insecta	Hemiptera	*Euscelidius variegatus*	NAT			8
Insecta	Hemiptera	*Geotomus punctulatus*	NAT	12	28	1
Insecta	Hemiptera	*Kelisia ribauti*	NAT	1		3
Insecta	Hemiptera	*Kleidocerys ericae*	NAT		11	2
Insecta	Hemiptera	*Megamelodes quadrimaculatus*	NAT	2		
Insecta	Hemiptera	*Monalocoris filicis*	NAT	1		
Insecta	Hemiptera	*Nabis pseudoferus ibericus*	NAT	6	3	22
Insecta	Hemiptera	*Nezara viridula*	INTR	7	5	46
Insecta	Hemiptera	*Nysius atlantidum*	END		2	116
Insecta	Hemiptera	*Orius laevigatus laevigatus*	NAT	6	8	210
Insecta	Hemiptera	*Oxycarenus lavaterae*	INTR		4	244
Insecta	Hemiptera	*Pilophorus confusus*	NAT	72	2	23
Insecta	Hemiptera	*Plinthisus brevipennis*	NAT	1		
Insecta	Hemiptera	*Pyrrhocoris apterus*	NAT		7	1
Insecta	Hemiptera	*Rhopalosiphum rufiabdominalis*	INTR	2		
Insecta	Hemiptera	*Saldula palustris*	NAT			4
Insecta	Hemiptera	*Scolopostethus decoratus*	NAT	26	12	
Insecta	Hemiptera	*Siphanta acuta*	INTR	10		
Insecta	Hemiptera	*Taylorilygus apicalis*	INTR	63	213	48
Insecta	Hemiptera	*Trigonotylus caelestialium*	NAT	21	36	76
Insecta	Hymenoptera	*Hypoponera eduardi*	NAT	35	9	1
Insecta	Hymenoptera	*Lasius grandis*	NAT	1587	672	881
Insecta	Hymenoptera	*Monomorium carbonarium*	NAT	224	237	315
Insecta	Hymenoptera	*Temnothorax unifasciatus*	NAT	13		4
Insecta	Hymenoptera	*Tetramorium caespitum*	NAT	99	33	17
Insecta	Lepidoptera	*Agrotis ipsilon*	NAT	1	2	
Insecta	Lepidoptera	*Aproaerema anthyllidella*	INTR	4		2
Insecta	Lepidoptera	*Autographa gamma*	NAT		5	
Insecta	Lepidoptera	*Blastobasis marrocanella*	NAT	1	6	3
Insecta	Lepidoptera	*Colias croceus*	NAT	10	13	2
Insecta	Lepidoptera	*Lampides boeticus*	NAT		3	
Insecta	Lepidoptera	*Mythimna unipuncta*	NAT	5	5	2
Insecta	Lepidoptera	*Oinophila v-flava*	INTR	11	33	1
Insecta	Lepidoptera	*Opogona sacchari*	INTR	1		3
Insecta	Lepidoptera	*Rhopobota naevana*	INTR		1	
Insecta	Lepidoptera	*Udea ferruginalis*	NAT	1		
Insecta	Microcoryphia	*Dilta saxicola*	NAT		2	2
Insecta	Neuroptera	*Hemerobius azoricus*	END		1	
Insecta	Odonata	*Sympetrum fonscolombii*	NAT		1	
Insecta	Orthoptera	*Conocephalus chavesi*	END	34	340	18
Insecta	Orthoptera	*Eumodicogryllus bordigalensis*	INTR	10	148	37
Insecta	Orthoptera	*Gryllus bimaculatus*	INTR	1	8	4
Insecta	Orthoptera	*Nemobius sylvestris*	INTR		2	
Insecta	Orthoptera	*Oedipoda caerulescens*	NAT			1
Insecta	Orthoptera	*Phaneroptera nana*	NAT	31	52	9
Insecta	Orthoptera	*Platycleis sabulosa*	NAT		11	16
Insecta	Orthoptera	*Trigonnidium cicindeloides*	INTR	4	1	6
Insecta	Phasmatodea	*Carausius morosus*	INTR	9		
Insecta	Psocoptera	*Atlantopsocus adustus*	NAT			3
Insecta	Psocoptera	*Bertkauia lucifuga*	NAT		1	
Insecta	Psocoptera	*Ectopsocus briggsi*	INTR	20	75	43
Insecta	Psocoptera	*Ectopsocus strauchi*	NAT	13	36	48
Insecta	Psocoptera	*Psyllipsocus ramburi*	INTR			1
Insecta	Psocoptera	*Trichopsocus clarus*	NAT	21	2	21
Insecta	Psocoptera	*Valenzuela burmeisteri*	NAT		3	
Insecta	Psocoptera	*Valenzuela flavidus*	NAT	13	56	53
Insecta	Thysanoptera	*Aeolothrips gloriosus*	INTR			4
Insecta	Thysanoptera	*Heliothrips haemorrhoidalis*	INTR		1	2
Insecta	Thysanoptera	*Hoplothrips corticis*	NAT		7	
Insecta	Zygentoma	*Ctenolepisma longicaudata*	INTR			1
		**Species Richness**		**130**	**148**	**130**
		**Abundance**		**4632**	**6461**	**4717**
